# Bibliographical

**Published:** 1867-10

**Authors:** 


### BIBLIOGRAPHICAL.

*A Dictionary of Medical Terminology, Dental Surgery, and The Collateral Seiences.* By CHAPIN A. HARRIS, M. D., D. D. S. Third Edition, carefully revised and enlarged, by FERDINAND J. S. GORGAS, M. D., D. D. S.

WE have just received a copy of this excellent work, and given it a cursory examination, and cannot do less than express our high appreciation of the manner in which the editor has performed his work.

The work is thoroughly revised, and "between two and three thousand new words have been added to the present edition, and additions and corrections made to the definitions of many others."

This work will be a valuable addition to the library of the Dentist, and indeed it cannot be complete without it. It will be a book of daily reference for both the student and practitioner. It will to a large extent supply the place of the medical dictionary, while at the same time, it presents all the words or terms of the Dental specialty. It will, we doubt not, very soon find its way into the library of every Dentist in the country, as well as be in the hands of every student.

